# Twenty years of the Fabry Outcome Survey (FOS): insights, achievements, and lessons learned from a global patient registry

**DOI:** 10.1186/s13023-022-02392-9

**Published:** 2022-06-20

**Authors:** Michael Beck, Uma Ramaswami, Elizabeth Hernberg-Ståhl, Derralynn A. Hughes, Christoph Kampmann, Atul B. Mehta, Kathleen Nicholls, Dau-Ming Niu, Guillem Pintos-Morell, Ricardo Reisin, Michael L. West, Jörn Schenk, Christina Anagnostopoulou, Jaco Botha, Roberto Giugliani

**Affiliations:** 1 SphinCS GmbH, Institute Clinical Science LSD, Hochheim, Germany; 2 Lysosomal Disorders Unit, Institute of Immunity and Transplantation, Royal Free London NHS Foundation Trust, University College London, London, UK; 3 Late Phase Solutions Europe AB, Täby, Sweden; 4 Johannes Gutenberg School of Medicine, University of Mainz, Mainz, Germany; 5 Department of Haematology, University College London, London, UK; 6 The Royal Melbourne Hospital, University of Melbourne, Parkville, VIC Australia; 7 Taipei Veterans General Hospital, Taipei, Taiwan; 8 Reference Centre for Hereditary Metabolic Disorders (MetabERN), Vall d’Hebron University Hospital, Barcelona, Spain; 9 Hospital Británico de Buenos Aires, Buenos Aires, Argentina; 10 Department of Medicine, Dalhousie University, Halifax, NS Canada; 11 Takeda Pharmaceuticals International AG, Zurich, Switzerland; 12 Department of Genetics, UFRGS, Medical Genetics Service, HCPA, Porto Alegre, Brazil

**Keywords:** Agalsidase alfa, Enzyme replacement therapy, Fabry disease, Cardiovascular outcomes, Renal outcomes

## Abstract

**Background:**

Patient registries provide long-term, real-world evidence that aids the understanding of the natural history and progression of disease, and the effects of treatment on large patient populations with rare diseases. The year 2021 marks the 20th anniversary of the Fabry Outcome Survey (FOS), an international, multicenter, observational registry (NCT03289065). The primary aims of FOS are to broaden the understanding of Fabry disease (FD), an X-linked lysosomal storage disorder, and to improve the clinical management of affected patients. Here, we review the history of FOS and the analyses and publications disseminated from the registry, and we discuss the contributions FOS studies have made in understanding FD.

**Results:**

FOS was initiated in April 2001 and, as of January 2021, 4484 patients with a confirmed diagnosis and patient informed consent have been enrolled from 144 centers across 26 countries. Data from FOS have been published in nearly 60 manuscripts on a wide variety of topics relevant to FD. Analyses of FOS data have investigated the long-term effectiveness and safety of enzyme replacement therapy (ERT) with agalsidase alfa and its effects on morbidity and mortality, as well as the benefits of prompt and early treatment with agalsidase alfa on the progression of cardiomyopathy and the decline in renal function associated with FD. Based on analyses of FOS data, ERT with agalsidase alfa has also been shown to improve additional signs and symptoms of FD experienced by patients. FOS data analyses have provided a better understanding of the natural history of FD and the specific populations of women, children, and the elderly, and have provided practical tools for the study of FD. FOS has also provided methodology and criteria for assessing disease severity which contributed to the continuous development of medical practice in FD and has largely improved our understanding of the challenges and needs of long-term data collection in rare diseases, aiding in future rare disease real-world evidence studies.

**Conclusion:**

FOS over the last 20 years has substantially increased the scientific knowledge around improved patient management of FD and continues to expand our understanding of this rare disease.

## Background

Disease registries and the real-world evidence they provide are highly valuable to physicians and patients. Registries increase the understanding of treatment effects and the natural history and progression of disease and allow the observation of large patient populations [1, 2]. Whereas randomized clinical trials are of short duration and test hypotheses, registries are of longer duration and generate hypotheses. Registry data, particularly for rare diseases, are increasingly important to government regulatory bodies and payers: the data extend the body of information that is available regarding long-term treatment effectiveness, the safety of medications, and health-related quality of life for patients. The year 2021 marked the 20th anniversary of the Fabry Outcome Survey (FOS), an international, multicenter, observational, physician-directed registry (NCT03289065) sponsored by Shire Human Genetic Therapies, Inc., a Takeda company [3, 4]. The primary aims of the FOS registry are to provide long-term data on the effectiveness and safety of enzyme replacement therapy with agalsidase alfa, broaden the understanding of Fabry disease (FD) and to improve the clinical management of affected patients [3, 4].

FD is an X-linked lysosomal storage disorder caused by more than 965 different mutations in the galactosidase alpha (*GLA*) gene that result in reduced or absent α-galactosidase A (α-Gal A) enzyme activity. This in turn leads to the accumulation of globotriaosylceramide (Gb3) [5–11]. Gb3 accumulation results in cell, tissue, and organ damage, leading to multisystem pathology typified by progressive renal and cardiovascular dysfunction; neuropathy; cerebrovascular events; ocular, dermatologic, gastrointestinal, and neuro-otologic manifestations; depression; and premature death [12–14]. Estimates of prevalence of FD range from approximately 1 in 117,000 to 1 in 37,000 live male births for classic FD and up to 1 in 1400 in some newborn screening projects when atypical FD variants are included [4, 15–18]. As an X-linked disease, FD had originally been considered as a disease mainly affecting male patients; however, female patients who are heterozygous for *GLA* mutations can also be affected, although onset is later and phenotypes are more variable [6, 13]. The variable clinical manifestation in female patients is attributed to many factors, including skewed X-inactivation; genetic variants; clonal expansion (which results in the secretion of the mature form of α-Gal A enzyme that is not readily taken up by cells); and somatic mosaicism resulting in an α-Gal A enzyme that is more susceptible to dephosphorylation in the plasma [19].

Enzyme replacement therapy (ERT) has been available for the treatment of FD since 2001 and is the standard of care [20]. Two ERT formulations are available currently [21–24]: agalsidase alfa (Replagal®, Shire Human Genetic Therapies AB, a member of the Takeda group of companies, Stockholm, Sweden, and first authorized 03 August 2001) [21, 22] and agalsidase beta (Fabrazyme®, Genzyme Corporation, a Sanofi company, Cambridge, MA, USA [23]; and Fabagal®, ISU Abxis Co, Ltd., Seoul, Republic of Korea [24]). Additionally, an α-Gal A pharmacological chaperone, migalastat (Galafold®, Amicus Therapeutics, Cranbury, NJ, USA) [25], can be used to treat certain patients with an amenable *GLA* mutation.

Patients with FD need life-long treatment that protects their organ function and improves survival. According to Wanner et al., “The aim of (Fabry disease) treatment is not only to slow or stop the progression of the disease and restore quality of life, but also to reverse Fabry pathology, minimizing the disease-associated morbidity and ultimately prolonging survival” [20]. There is a need for long-term effectiveness and safety data for FD treatments from a real-world setting. One way to obtain these data is by following patients undergoing routine treatment with available therapies as part of well-designed disease registries [1]. The inherent nature of long-term, observational patient registries makes them ideal for the assessment of treatment effectiveness in rare diseases in clinical practice [2]. Registries may include a more diverse range of patients regarding age, sex, disease severity, comorbidities, concomitant medications, or treatment than randomized clinical trials by having less strict inclusion criteria [1].

Considering the value of a registry in understanding disease and treatment management in the real world and the significance of having over 20 years of data from a long-term, rare disease registry, we review here the publications that have been developed on the basis of FOS registry data and discuss the contributions FOS has made to the scientific knowledge of FD.

## Design, history, and evolution of FOS

FOS was established in April 2001 by TKT Europe-5S AB, under the direction of a European board and an executive committee, each comprising a panel of elected physicians. The objectives were to collect data on long-term clinical outcomes and safety, and to collect information on the rate of disease progression initially in European patients with FD who were untreated or treated with agalsidase alfa. From the outset, FOS data were used strictly for scientific aspects and regulatory requirements. The design of FOS has been described in detail in previous publications and has enabled the pooling of data from multiple centers to obtain sample sizes large enough for the evaluation of outcomes in treated and untreated patients [3, 4, 12, 26]. Patients or their caregivers provide consent for their data to be included via signed informed consent forms. Data are collected on a variety of key parameters, including—but not limited to—safety, cardiovascular, renal, cerebrovascular, and neurological evaluations, pain assessment, treatment, and quality of life (QoL). More recently (January 2017), information on biomarkers has been added (e.g., globotriaosylsphingosine in urine and blood), and there are now separate questionnaires for pediatric patients. From 2005, a number of features designed to capture more comprehensive data were incorporated into FOS, including a focus on larger specialist centers (with ≥ 20 patients) and the collection of data for a number of core FD-related variables covering both clinical and QoL-related parameters (in addition to comprehensive health checks) [2, 4].

On 26 March 2006, FOS was merged with the Fabry International Research Exchange (FIRE) database. FIRE, established in 2003 by Transkaryotic Therapies Inc, was a registry in non-European countries that was open to patients on ERT with a confirmed diagnosis of FD. The primary purpose of the FIRE registry was to document the natural history of FD, document changes in disease progression resulting from long-term ERT, provide a basis for developing treatment guidelines, and provide data that would lead to the development of better treatments.

The FOS protocol was subsequently amended on 29 June 2016 (Protocol Amendment 4) to become a disease registry so that any patient with FD, regardless of treatment, could be enrolled in FOS. This amendment enabled the collection of data from patients who switched treatments (e.g., from agalsidase alfa to agalsidase beta and/or migalastat) during the management of their disease.

FOS is directed by the FOS Steering Committee and representatives of the sponsor, which enable the smooth operation of the registry and ensure the scientific accuracy and appropriateness of all research concepts and proposals for data analyses and publications. The FOS Steering Committee includes FOS investigators who represent the geographical regions where the registry operates, and a patient organization delegate. The Steering Committee provides guidance on the overall direction of the registry by raising topics of scientific interest to be studied, endorsing data analyses and topics for publication, overseeing data quality and completeness, overseeing the functioning and activities of the Task Forces, and representing FOS at scientific meetings. FOS Task Forces were established to address specific topics. Task Force members are physicians or researchers with a particular expertise and interest in the relevant areas, such as nephrology, neurology, or cardiology, and they are responsible for all activities related to conducting and publishing in-depth analyses of FOS data in their field of interest.

### Achievements of FOS

Since the first patient enrolled in FOS in 2001, the number of participating centers and countries has increased steadily, reaching 4484 patients with confirmed patient informed consent enrolled in FOS from 144 centers across 26 countries as of January 2021. In line with its original aims, FOS has contributed to the overall knowledge of FD, including a better understanding of the morbidity and mortality relating to the various organ systems affected; has provided methodology and criteria for grading disease severity; has defined the manifestations of FD in women, children, and the elderly; has improved patient management and led to earlier treatment; and has improved knowledge of FD genetic variants that result in late-onset versus classic forms of the disease.

**Fig. 1:**
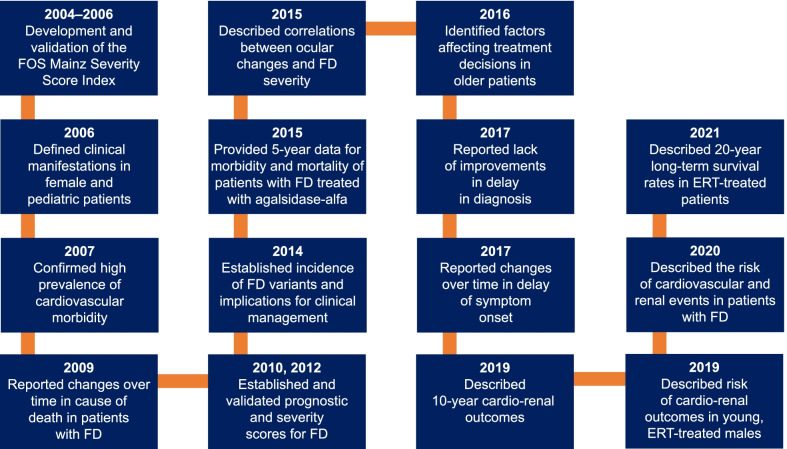
Highlights of FOS publications and their contribution to the understanding of Fabry disease. *ERT* enzyme replacement therapy, *FD* Fabry disease, *FOS* Fabry Outcome Survey

As of January 2021, nearly 60 manuscripts utilizing data from FOS have been published on a wide variety of topics relevant to FD (Fig. 1). Instruments created from FOS include the FOS Mainz Severity Score Index (FOS-MSSI) [27, 28], the Fabry International Prognostic Index (FIPI) [29], and the Fabry-specific Pediatric Health and Pain Questionnaire (FPHPQ) [30]. FIPI is a prognostic severity scoring index that uses early symptoms of FD to differentiate patients with varying probabilities of experiencing a clinically significant event [29]. The FPHPQ is a quantitative assessment of patient-reported FD symptoms in children used to monitor disease progression and treatment effects [30]. The Mainz Severity Score Index (MSSI) [28] is used to score disease severity in FD. However, FOS uses a signs and symptoms checklist that produces dichotomous variables (i.e., yes or no), which are incompatible with scoring by the original MSSI [31]. The MSSI was therefore modified to accommodate FOS data, resulting in the FOS-MSSI [27, 28, 31]. The FOS-MSSI has sections relating to general, neurological, cardiovascular, and renal disease manifestations [31]. Signs and symptoms are weighted in accordance with the data collected in FOS, and the total score for disease severity obtained may be categorized as mild (≤ 18), moderate (19–38), or severe (> 38)[6]. As such, the FOS-MSSI is used to give a description of the cumulative disease burden of FD (in contrast to the original MSSI) [6, 27, 28, 31].

### Fabry disease characteristics

**Fig. 2:**
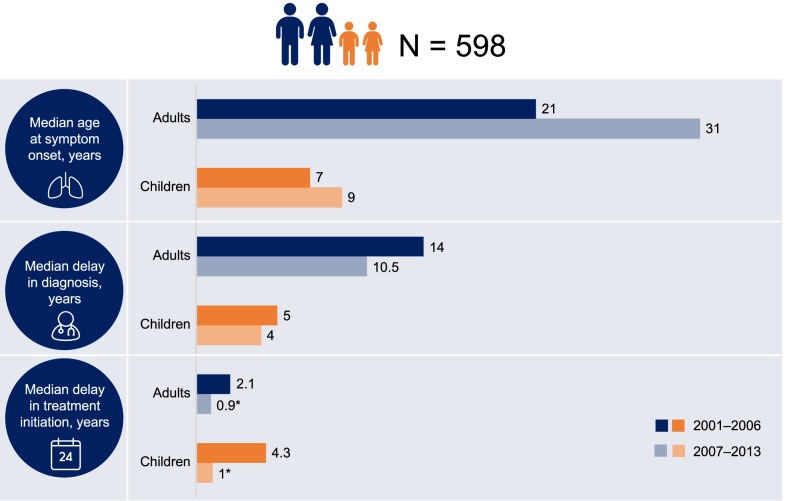
Summary of Fabry Outcome Survey data relating to age at onset, diagnosis, and treatment initiation for adults and children. *p < 0.001 vs. earlier period. Data from Reisin et al. [32]

Analyses of FOS data have provided insight into the natural history of FD (classic and variants) and into the impact of treatment on disease progression in several affected organ systems and populations. FD affects neurologic, dermatologic, renal, cardiac, auditory, vascular, cerebrovascular, gastrointestinal, and ocular organ systems [12]. Signs and symptoms can include neuropathic pain, angiokeratomas, proteinuria, angina and dyspnea, gastrointestinal symptoms, cerebrovascular events, or tinnitus and vertigo. Many FOS publications raise awareness of the delays in receiving a diagnosis after the onset of symptoms. Among the first publications from FOS, an analysis of baseline (FOS entry) data for 366 patients from 11 different countries illustrated the similar occurrence of FD symptoms in hemizygous male and heterozygous female patients [12]. The same analysis also showed substantial delays between symptom onset and diagnosis in male and female patients (13.7 and 16.6 years, respectively) [12]. Approximately 25% of FOS patients were misdiagnosed with rheumatological diseases, neuropsychological diseases, orthopedic diseases, and “others”, which included renal disease and coronary artery disease. A 2017 FOS analysis in 598 patients with FD found that the delay between symptom onset and diagnosis did not significantly improve between 2001–2006 and 2007–2013, although the median delay between diagnosis and treatment initiation decreased to approximately 1 year in children and 0.9 years in adults in the period 2007–2013 (Fig. 2) [32].

### Prognosis

FOS data have been used to explore prognostic factors related to disease severity. The availability of data from FOS for a large number of patients (n = 1438) enabled the use of event-free survival for cardiovascular, renal, and neurologic endpoints to develop a validated prognostic scoring system for FD. This scoring system, called FIPI, can be used to differentiate patients based on the likelihood and timing of progression, and is therefore a useful aid for patient counseling and disease management [29]. An analysis exploring genotype–phenotype relationships found a highly significant correlation between age at entry into FOS and severity score, as well as between age at entry into FOS and the number of organs affected in male patients with missense mutations, suggestive of important associations between genotype and disease severity [33].

### Long-term effectiveness of ERT

The long-term safety and effectiveness of ERT in pediatric and adult patients is also supported by data from FOS [34–40]. Overall, male and female patients had similar responses to agalsidase alfa, suggesting that there should be no difference in access to treatment if treatment criteria are met [41]. There is evidence, however, that there are sex disparities in initiating treatment with agalsidase alfa. This was illustrated by an analysis of FOS data for patients in Spain, which found that female patients with disease characteristics likely to benefit from ERT were less likely to receive treatment than male patients [42], an observation that was also seen in a German, non-FOS study of 261 female patients with FD [43]. These results also show discordance with consensus recommendations on when to start treatment respective to each country, indicating that better adherence to treatment guidelines might lead to better outcomes for patients with FD in various countries.

### Benefits of prompt and early treatment with ERT

FOS data have shown that not only does ERT benefit patients with FD by attenuating the progression of renal disease and cardiomyopathy, but also that prompt treatment reduces the risk of cardiovascular and renal events such as heart failure or dialysis, regardless of FD type (i.e., late-onset vs. classic FD) [44]. A FOS analysis that included 1374 patients (172 promptly treated, 1202 delayed; males 807, females 567) based on time from symptom onset and 2051 (1106 promptly treated, 1045 delayed; males 1130, females 921) based on time from diagnosis showed that in the overall population, prompt ERT initiation was associated with significant benefits in the reduction of cardiovascular events—both since symptom onset (hazard ratio [HR] = 0.62; *p* < 0.001) and since diagnosis (HR = 0.83; *p* < 0.003) [44]. These benefits occurred in both male and female patients with FD (female vs. male: symptom HR = 0.83, *p* = 0.018; diagnosis HR = 0.82, *p* = 0.003). Further, analysis by age at symptom onset showed that the beneficial effect of prompt initiation was more profound in patients ≤ 20 years of age at symptom onset [45]. An analysis of 560 male patients with FD showed the benefits of starting treatment in childhood or early adulthood. Starting ERT at age ≤ 18 years attenuated the progression of renal disease and cardiomyopathy, whereas patients receiving ERT for similar durations who started treatment as adults (> 18 years) had statistically significant worsening in estimated glomerular filtration rate (eGFR) if started at age > 18 years to ≤ 30 years, and in eGFR, proteinuria, and left ventricular mass index (LVMI) if started at age > 30 years [45]. A different FOS analysis of LVMI and eGFR subgroups (LVMI, n = 560; eGFR, n = 1093) in male and female patients with FD showed that the risks of cardiovascular or renal events were significantly higher (both log-rank *p* < 0.0001) in patients with left ventricular hypertrophy (LVH) and in patients with eGFR < 90 mL/min/1.73 m^2^ (“abnormal eGFR”) at baseline. The patients who had LVH or low eGFR at baseline were significantly older at symptom onset and diagnosis than those patients with normal LVMI or eGFR at baseline (*p* < 0.001) [39]. A FOS analysis of data from pediatric patients also demonstrated that early treatment with ERT reduced FD symptoms such as pain, vomiting, constipation, and diarrhea at both 12 and 24 months from baseline. In children, baseline renal and cardiac parameters were stable and remained stable with treatment [35]. These FOS analyses together indicate that early, prompt treatment with ERT according to treatment guidelines, before irreversible organ damage has occurred, can help preserve organ function and protect patients from organ damage caused by FD.

### Cardiovascular manifestations

Analyses of data from FOS have facilitated better understanding of cardiovascular manifestations and long-term cardiovascular outcomes in patients with FD. The high prevalence of cardiovascular morbidity among patients with FD was confirmed in patients enrolled in FOS, with a greater cardiovascular disease burden observed in male patients than in female patients. Cardiovascular manifestations included dyspnea, heart failure, angina, palpitations, arrhythmia, and syncope [46]. A long-term analysis using deconstructed composite events showed that cardiac events occurred at younger ages than renal or cerebrovascular events, and that male patients were more likely than female patients to experience cardiac events at a younger age [36, 38, 47, 48]. In patients treated with agalsidase alfa over 10 years, mean LVMI increased slightly in patients with LVH at baseline, compared with no increases in patients with no LVH at baseline (Table 1) [37, 38].

**Table 1:** Effect of ERT on clinical outcomes in FOS patients

System	Effect of ERT	Publication
Cardiovascular	Mean LVMI increased slightly but significantly over 10 years in patients with LVH at baseline, compared with no increases in patients with no LVH at baseline	Kampmann et al. [37] Ramaswami et al. [38]
	LVH was a significantly better predictor of long-term cardiovascular outcomes than normal LVMI in adults at the start of ERT	Feriozzi et al. [39]
	Patients with a low eGFR at baseline had a significantly higher risk for a cardiovascular event compared to those with normal eGFR	
	Cardiovascular structure and function improved, and renal function stabilized during treatment, although LVMI was only reduced significantly in female patients	Hughes et al. [41]
	Composite morbidity events occurred at an older age compared with published findings in untreated patients with FD	Beck et al. [47]
Renal	Stabilization of eGFR in female patients 10-year longitudinal data: Reduction in the annual rate of eGFR decline was smaller in treated male patients than expected in untreated patients	Mehta et al. [26] Feriozzi et al. [52] Feriozzi et al. [53] Ramaswami et al. [38]
	Patients with hyperfiltration (eGFR > 130 mL/min/1.73m^2^) at baseline attained an eGFR within the accepted normal range (> 90 mL/min/1.73m^2^) with 5 years of starting ERT	Mehta et al. [26]
	Renal function remained stable in 20 patients with FD who had undergone kidney transplantation and who had received ERT	Cybulla et al. [56]
	Renoprotective effect of ERT is independent of the type of FD mutation	Cybulla et al. [40]
Morbidity and Mortality	Most common cause of death shifted from renal disease (pre-2001) to cardiovascular disease (post-2001)	Mehta et al. [48]
	Median survival for male ERT-treated patients at 5 years was 17.5 years longer than for untreated male patients	Beck et al. [36]
	Probability of a composite morbidity event for ERT vs. placebo was ~ 16% vs. ~ 45%	Beck et al. [36]
Auditory	Stabilized and/or decreased the progression of hearing loss in FD	Hajioff et al. [62]
Gastrointestinal	Prevalence of abdominal pain in male patients and children and prevalence of diarrhea was significantly reduced after 1 year of ERT	Hoffmann et al. [63, 64]
Pain	Improved patient pain levels as assessed by the Brief Pain Inventory after 2 years of ERT	Hoffmann et al. [67]

*eGFR* estimated glomerular filtration rate, *ERT* enzyme replacement therapy, *FD* Fabry disease, *FOS* Fabry Outcome Survey, *LVH* left ventricular hypertrophy, *LVMI* left ventricular mass index

Another analysis showed that patients treated with agalsidase alfa had a negligible annualized rate of change in LVMI. The mean annual rate of LVMI change was 0.33 g/m^2.7^/y in males and 0.48 in females, compared with LVMI increasing at a rate of 4.07 in untreated males and 2.31 in untreated females [35]. Thus, treated patients with LVH at baseline are expected to have a smaller increase in LVMI compared to untreated patients with LVH at baseline, which is a further rationale for prompt treatment with regular follow-up and tests to evaluate disease progression [36]. Furthermore, a recent analysis of FOS data found that LVH present at the start of ERT was a risk factor for cardiovascular events. Moreover, the subgroup with a low eGFR at baseline also had a significantly higher risk for a cardiovascular event (*p* < 0.001; Table 1) [39]. A comparison of outcomes after 4 years’ FD treatment with ERT in female patients (n = 78) and male patients (n = 172) in FOS found that cardiovascular structure and function improved and renal function stabilized during treatment, although LVMI was only reduced significantly in female patients (Table 1) [41].

FOS has also enabled the study of cardiovascular events in patients with atypical FD (e.g., patients with *ISV4* mutations) [9, 10]. Compared with patients with classic FD, male (but not female) patients with *ISV4* mutations were significantly older when their LVH was initially diagnosed, but no age differences were apparent for other cardiovascular diagnoses [10]. Endomyocardial biopsy data for 30 patients with *ISV4* mutations enrolled in FOS showed that a longer duration of ERT was associated with lower Gb3 accumulation and smaller cardiomyocytes, suggesting a possible impact of ERT on the pathophysiology of the disease. The correlation between Gb3 accumulation and LVMI was statistically significant, but moderate, likely because of the small number of biopsies evaluated [9]. In addition, retrospective study data showed that there is a reduced risk of thromboembolic events in patients with FD on ERT [49].

Uncontrolled hypertension was found to be highly prevalent in FOS (57% of male patients, 47% of female patients; n = 391), and both systolic and diastolic blood pressure decreased significantly after 2 years of ERT [50]. Hypertension has been linked with decline in renal function in long-term analyses of FOS data [38, 39].

Anemia was recorded in 20% of female patients and 47% of male patients (n = 345), and appeared to be associated with decreased eGFR and heart failure—both risk factors for anemia—as well as with inflammation (elevated C-reactive protein). However, although ERT may have positive effects on the cardiovascular manifestations of FD, there is no evidence to date for an effect of ERT on anemia [51].

### Renal manifestations

Data from FOS have provided much-needed insights into renal function in patients with FD, and the impact of long-term ERT on renal outcomes (Table 1). An analysis of FOS data from 366 patients with FD revealed that, prior to ERT start, 50% of patients reported renal events (i.e., proteinuria, dialysis, end-stage renal disease, or renal transplant) [12]. The benefit of long-term ERT for up to 10 years in patients with FD-related nephropathy, where baseline eGFR was ≥ 30 ml/min/1.73^2^, was later confirmed using FOS data, with stabilization of eGFR achieved in female patients, and a reduction in the annual rate of eGFR decline in male patients (Table 1) [26, 38, 52, 53]. After 5 years of treatment with ERT, 12 of 14 patients (86%) who had hyperfiltration (eGFR > 130 mL/min/1.73 m^2^) at baseline attained an eGFR within the accepted normal range (> 90 mL/min/1.73 m^2^) [26]. Additionally, in long-term analyses with up to 10 years’ follow-up, baseline proteinuria > 1 g/24 h was found to have a negative influence on renal function over time [38, 39]. Twelve-year data from additional long-term analyses of male FOS patients found that ERT with agalsidase alfa may be associated with potential renoprotective effects, leading to the stabilization of renal function relative to the expected decline in patients with FD [40, 54, 55]. A greater annual rate of change in proteinuria was observed in a group of patients with FD, including patients previously receiving angiotensin receptor blockers or angiotensin-converting enzyme inhibitors, and high renal involvement (proteinuria > 0.5 g/24 h) at baseline versus a group of patients with FD and low renal involvement (proteinuria ≤ 0.5 g/24 h) at baseline, confirming the role of proteinuria as a risk factor for renal impairment [38, 40, 54, 55]. These observations should be considered in the context of the natural history of FD as described in the literature. A retrospective chart review of 279 male untreated patients examined between 1944 and 2002, with only approximately one-third of these patients receiving angiotensin receptor blockers or angiotensin-converting enzyme inhibitors, showed more rapid loss of renal function with proteinuria at baseline [38]. Renal function remained stable in 20 patients with FD who had undergone kidney transplantation and who had received agalsidase alfa; however, further data are required to evaluate the long-term renoprotective effects of ERT following transplantation (Table 1) [55, 56].

Long-term FOS data confirmed an association between renal and cardiovascular outcomes, with greater declines in renal function over time apparent in patients with baseline arterial hypertension compared with those without [38, 39, 53], and greater overall risk of experiencing a cardiovascular event in patients with poor renal function at baseline compared with patients with eGFR at or near the reference range [39]. Ultimately, FOS data suggest that ERT should be administered at an early stage of disease to reduce FD progression and protect patients from organ damage.

### Cerebrovascular manifestations

The incidence and prevalence of cerebrovascular events in patients with FD were identified in FOS studies for male versus female patients and classic versus atypical FD phenotype. Cerebrovascular events (primarily transient ischemic attacks and ischemic strokes) are an important cause of morbidity and mortality in patients with FD, although incidence rates vary. Cerebrovascular events occurred in 11.1–25% of male patients and 15.7–21% of female patients enrolled in FOS [48, 57], although male patients experienced these events at a younger age than female patients (mean 39.2 and 51.4 years, respectively) [48]. The number of ischemic lesions found on brain magnetic resonance imaging was similar in classic and atypical (*ISV4* mutation) FD and much higher than in the general population, with some differences in location in a study of FOS participants in Taiwan [58]. While the evidence to support a direct effect of ERT on cerebrovascular events is still controversial, the likelihood of indirect effects due to improved cardiovascular and renal function has been suggested [57].

### Ocular, auditory, gastrointestinal, and dermatologic manifestations

FOS analyses have shown that ocular, auditory, gastrointestinal, and dermatologic manifestations of FD may be important signs of disease severity and can affect patient QoL. The most common ocular signs of FD in FOS were cornea verticillata, tortuous conjunctival/retinal vessels, and posterior spoke-like cataracts [59, 60]. The high prevalence of ocular signs and symptoms in children, affecting 54.5% of girls (n = 101) and 47.3% of boys (n = 131) with FD, suggests they could be a valuable diagnostic tool in this age group. Children and adults with ocular findings also had higher median age–adjusted FOS-MSSI scores than those without, suggestive of an association with more severe FD in children and adults [59, 61]. The prevalence of cornea verticillata was also significantly higher in patients with null or missense *GLA* mutations, compared with those with the *p.N215S* or mild missense mutations [61].

FOS studies have shown that ERT can stabilize and/or decrease the progression of hearing loss in FD. Analysis of FOS data determined that although there were no changes in patients with normal hearing or with severe hearing loss at baseline, hearing thresholds improved significantly by 4‒7 dB across most frequencies for those with mild or moderate hearing loss at baseline, suggesting that agalsidase alfa can stabilize or possibly improve hearing in this small number of patients who have not progressed to severe hearing loss, however further studies are required [62].

FOS analyses have shown that ERT can improve gastrointestinal symptoms in patients with FD. Among the patients enrolled in FOS (n = 342), gastrointestinal symptoms were reported for 54.2% of females and 48.9% of males, and included abdominal pain (32.5%), diarrhea (20.5%), constipation (13.5%), and nausea (12.3%) and vomiting (6.7%) [63, 64]. Abdominal pain was present in 49.3% of children and, like diarrhea, was more frequent in children than adults [35, 63, 64]. After 1 year of ERT, the prevalence of abdominal pain in male patients and children was significantly reduced, as was the prevalence of diarrhea in children, with no child reporting diarrhea as a new symptom; results were similar after 2 years (Table 1) [63, 64].

Dermatologic manifestations are common in FD and were found in 78% of male patients and 50% of female patients in an analysis of early FOS data [12]. Among patients enrolled in FOS, angiokeratomas were the most common dermatologic manifestation and occurred more frequently (66% vs. 36%) and at a younger age (mean 17.9 vs. 29.1 years) in male versus female patients [12, 65]. Hypohydrosis was the next most common dermatologic symptom, occurring in 53% of male patients and 28% of female patients [65], whereas hyperhidrosis, although less common overall, was more prevalent in female patients (11.9%; n = 369) than male patients (6.4%; n = 345) with FD [66]. The presence of cutaneous vascular lesions in the skin was associated with more severe FD with major organ involvement; however, there is no clear evidence to support an effect of ERT on skin lesions [65] or other dermatologic issues [66].

### Pain and QoL

FOS has improved the understanding of how FD affects patient QoL. An analysis of a cohort of 120 FOS patients from the United Kingdom found that baseline health-related QoL scores (EuroQol 5 Dimension [EQ-5D] utility scores) were significantly below those of the general UK population, with no differences between male and female patients; however, significant improvements in QoL were achieved after 1 year of ERT, reaching scores similar to the general population [67]. Improvements in QoL were maintained after 5 years of ERT [26]. A key factor in the improvement in health-related QoL scores was change in the EQ-5D pain-related dimension score, underlining the impact of pain on QoL in FD [26, 67]. In addition to the QoL findings, a separate analysis found that following 2 and 5 years of ERT, patients in FOS reported that their pain had improved significantly from baseline as assessed by the Brief Pain Inventory (Table 1) [26, 67].

### Morbidity, mortality and causes of death

The availability of ERT, beginning in 2001, has brought about changes in the natural course of FD, not least an impact on life expectancy and causes of death. Among both male and female patients enrolled in FOS who received ERT, the most common cause of death was cardiovascular disease (34% of male patients and 57% of female patients) [48]. In contrast, the majority of deaths occurring before 2001 were attributed to renal disease, suggesting that ERT has contributed to a change in the relative importance of renal and cardiovascular disease in patients with FD [48]. Data from FOS suggest that the estimated median survival for male patients with FD treated with ERT for 5 years was 77.5 years, compared with 60 years for untreated male patients (Table 1) [36]. Composite morbidity events (including death) also occurred at an older age in FOS patients receiving ERT compared with published findings in untreated patients with FD [47, 55]. After 24 months, the probability of a composite morbidity event was approximately 16% in the ERT cohort overall compared with approximately 45% overall for the placebo group [36]. A survival analysis of 2251 FOS patients found that 157 patients (7.0%) treated with agalsidase alfa died through the whole 20-year study period. The median (quartile 1, quartile 3) survival time from baseline was 6.30 (3.62, 10.13) years. Overall, Kaplan–Meier probability estimates of survival for FOS patients treated with agalsidase alfa for 10, 15, and 19 years were 0.917, 0.843, and 0.700, respectively (log-rank *p* = 0.0003). Survival rates were also significantly higher for females compared with males at 15 years (log rank *P* = 0.0147) and overall (log rank *P* = 0.0213) [68].

### Manifestations in children, women, and the elderly

FOS analyses have also been performed on data from pediatric, adult female, and elderly cohorts, and have demonstrated the value of monitoring FD in these groups. Manifestations of FD may begin in childhood. The mean age of symptom onset in male and female adult patients was found to be 10.9 and 22.6 years of age, respectively. In children, median age of onset in male and female patients was shown to be 6.7 years of age and 7.8 years of age, respectively [12, 32, 69]. An analysis of data for 82 children enrolled in FOS found that the most frequent early manifestations of FD were neurologic (e.g., acroparesthesia, altered temperature sensitivity) and gastrointestinal symptoms, observed in 80% and 60% of children, respectively [69]. More than 40% of children also had auditory and dermatologic signs and symptoms (e.g., tinnitus, vertigo, angiokeratoma, and fatigue). Although the median age at symptom onset in this study was 6.7 years in boys and 2–5 years later in girls, symptoms were present at similar frequencies, and the mean time to diagnosis was approximately 3 years [69]. A 2011 retrospective chart review of eight children (mean age of 5.0 years) who began agalsidase alfa treatment at ≤ 7 years of age (mean duration of treatment 4.2 years) found that with agalsidase alfa treatment, mean eGFR was within the normal range at baseline and remained normal [34]. A more recent FOS analysis in 2020 found that in patients starting ERT in childhood or early adulthood, renal and/or cardiac disease progression was attenuated compared with adults > 30 years old [45]. For the 151 patients who were ≤ 18 years of age, mean annual rates of change in eGFR, proteinuria, and LVMI were stable and remained stable with agalsidase alfa treatment. However, patients who were > 18 years to < 30 years of age showed significant yearly deterioration in eGFR over the follow-up period (*p* < 0.001), and patients > 30 years of age showed significant yearly deterioration in eGFR, proteinuria, and LVMI (*p* < 0.001 for each), suggesting the value of initiating treatment early to protect from end-organ damage and attenuate disease progression [45].

Three FOS publications have specifically described FD in female patients. Among 303 female patients enrolled in FOS, the most common symptoms were neurologic (77%) and cardiovascular (59%), with renal signs and symptoms present in 40% [70]. Neurologic features appeared at a mean age of 16 years, cardiac features at 34 years, and renal involvement at 37 years. FOS data identified geographic differences in disease severity among female patients with FD, with female patients in northern European countries having significantly higher FOS-MSSI scores, representing more severe disease, than those living in southern European countries, although the extra-genetic or epigenetic (e.g., dietary) factors involved have not been identified [71]. These results, however, should be interpreted with caution because it is possible that the awareness or interpretation of disease (i.e., pain) in female patients differs in northern and southern European countries. A FOS analysis of reproductive and pregnancy-related outcomes showed that age at menarche and age at menopause were similar between never-treated female patients with FD and agalsidase alfa–treated female patients with FD [72]. Among those female patients with FD who completed a pregnancy questionnaire, 91.3% of 23 pregnancies in 21 female patients treated with agalsidase alfa versus 96% of 75 pregnancies in 52 never-treated female patients had a normal outcome; spontaneous abortion did not occur in any of the female patients treated with agalsidase alfa [72]. Further, no significant differences in any of the tested renal and cardiac parameters were observed after pregnancy, regardless of treatment status [72].

A limited number of analyses have been conducted on outcomes in elderly patients in FOS. A 2016 analysis of elderly patients in FOS showed that although FOS patients aged ≥ 75 years had more severe cardiomyopathy and a higher prevalence of hypertension, and lower eGFR than those aged 18‒49 years, a smaller proportion were being treated (50% vs. 64.6%). It was suggested that this could be due to a relatively milder disease burden in the older age group or to patient and/or physician reluctance to start or continue ERT in older patients [73]. In a subsequent preliminary analysis of FOS patients aged ≥ 65 years, eGFR and LVMI were significantly worse in elderly patients compared with other patients treated with agalsidase alfa, supporting the continuation of ERT in elderly patients [74].

### FOS findings in line with other real-world data

Data from FOS are further supported by findings from the Fabry Registry, a global observational research platform for FD, where most patients who died from FD were reported to have serious cardiac and renal dysfunction [75]. Furthermore, cardiovascular events were reported in more male than female patients [76] and were the most common type of initial clinical presentation in male and female with FD in Latin America [77]. Findings from this registry showed that initiation of ERT at a younger age reduced the risk for cardiovascular events [78] and/or renal dysfunction [79, 80]. However, despite ERT, patients with advanced FD continued to be at risk for serious cardiac or renal events [81–85]. Other country-specific registries also provided broadly similar data to FOS [86–90].

## Key learnings and challenges

Since its inception, FOS has contributed significantly to the understanding of FD, including the effects of ERT on late-onset versus classic FD. Further, ongoing analyses of FOS data have highlighted the changing landscape of FD over time. FOS publications have putatively increased physicians’ awareness of FD and perhaps thereby shortened delays in diagnosis. An update of the analysis of delays between symptom onset and diagnosis in FD is warranted. Additionally, analyses of biomarkers, documented mutations, genotype-phenotype, cerebrovascular events in FD and the impact of ERT on outcomes in elderly patients are still being considered for the future. However, several challenges have been encountered throughout the duration of FOS, many of which are related to the nature of registries, although others are specific to FOS. The quality of registry data observed in FOS is determined—and limited—by the quantity and quality of the information inputted by participating physicians. As data are collected during routine clinical practice, with variation in both the frequency of visits and the assessments carried out at each visit, data may be incomplete, resulting in low patient numbers for certain parameters and subgroups. Further improvements in data completeness by focusing on core variables are therefore vital. The most notable missing data in FOS are for genotype, owing to changes in patient informed consent documentation for these data. Also, baseline characteristics of treated and untreated patients in FOS are not comparable, as mildly affected untreated patients are switched to treatment as their disease becomes more severe, and therefore there is a need to use a historical comparator group instead of an internal comparator group in certain FOS analyses, which also creates challenges in interpreting results. Differences in diagnostic and treatment practices among regions and countries have also been a challenge with FOS.

## Looking to the future

Because FD is a chronic condition, patients may require long-term treatment throughout adulthood beyond the parameters studied previously. Findings from FD registries may be used to inform treatment guidelines, including establishing the ideal timing of treatment initiation, identifying novel biomarkers for better disease management, or helping to formulate management recommendations. Real-world data suggesting that female patients with FD may be under-treated [42] could support changes in medical practice that ensure female patients with FD receive ERT when appropriate. Future studies with larger sample sizes would be valuable to better understand atypical FD subtypes, such as those described for the mutations *IVS4* and *p.Ala143Thr* [8–10, 58]. As further developments are made in the diagnosis and management of patients with FD, FOS and other FD registries will need to be adapted to ensure they capture the most relevant data. For example, the addition of a free-text field in the FOS database would enable the collection of data regarding the effectiveness of new treatments or information regarding signs and symptoms that have not yet been observed or described. Patient-reported outcomes are increasingly recognized for their value in patient management, and more emphasis on these measures in future studies is needed. In the future, new technologies could enable faster, more efficient data aggregation from electronic medical records, as well as from other sources such as smartphone app-based patient-reported outcome questionnaires or mobile data-logging devices. More powerful data processing and artificial intelligence systems could potentially enable the extraction of uniform data for a particular parameter that may have originally been recorded in different formats. These efforts would aid data aggregation and analysis by making data more searchable, accessible, and extractable. In addition, as technology allows healthcare to become more patient centric, patients are increasingly keen to maintain control over their personal data. Development of block chain technology may allow increased security, privacy, and interoperability of health data. This technology could provide a new model for health information exchanges by making electronic medical records more efficient and disintermediated. Future registry analyses in FD should explore the lifetime impact of ERT in specific populations (e.g., patients receiving a kidney transplant), seek to develop better tools and biomarkers for diagnosis and the determination of FD severity, and further characterize FD mutations and their relationship with specific disease phenotypes. Ongoing efforts to better understand the natural history and symptoms of atypical FD subtypes are essential.

## Conclusions

The data collected in FOS over the last 20 years have increased our understanding of the natural history of FD. FOS analyses have contributed to the body of FD knowledge by evaluating the long-term effectiveness and safety of ERT with agalsidase alfa and the impact of ERT not only on survival but also on the cardiovascular and renal manifestations of FD. FOS analyses have also provided better understanding of the specific issues faced by women, children, and elderly patients with FD, and have provided practical tools for the study of FD.

## Data Availability

The datasets, including the redacted study protocol, redacted statistical analysis plan, and individual participants’ data supporting the results reported in this article will be made available within 3 months from initial request to researchers who provide a methodologically sound proposal. The data will be provided after its de-identification, in compliance with applicable privacy laws, data protection, and requirements for consent and anonymization.

## Abbreviations

α-Gal A: α-Galactosidase A
eGFR: Estimated glomerular filtration rate
EQ-5D: EuroQol 5 Dimension
ERT: Enzyme replacement therapy
FD: Fabry disease
FIPI: Fabry International Prognostic Index
FIRE: Fabry International Research Exchange
FOS: Fabry Outcome Survey
FOS-MSSI: FOS Mainz Severity Score Index
FPHPQ: Fabry-specific Pediatric Health and Pain Questionnaire
Gb3: Globotriaosylceramide
GLA: Galactosidase alpha
HR: Hazard ratio
LVH: Left ventricular hypertrophy
LVMI: Left ventricular mass index
MSSI: Mainz Severity Score Index
QoL: Quality of life
